# Supplementary material for: Effects of Trichoderma harzianum combined with Phanerochaete chrysosporium on lignin degradation and humification during chicken manure and rice husk composting
Source: Front Microbiol. 2025 Feb 28;16:1515931. doi: 10.3389/fmicb.2025.1515931 (PMC11906335; doi:10.3389/fmicb.2025.1515931)
Supplement: Supplementary file 1 [file Data_Sheet_1.zip › 6.Function/CKF_HF/FunGuild/l1/CKF_vs_HF_STAMP.pdf]

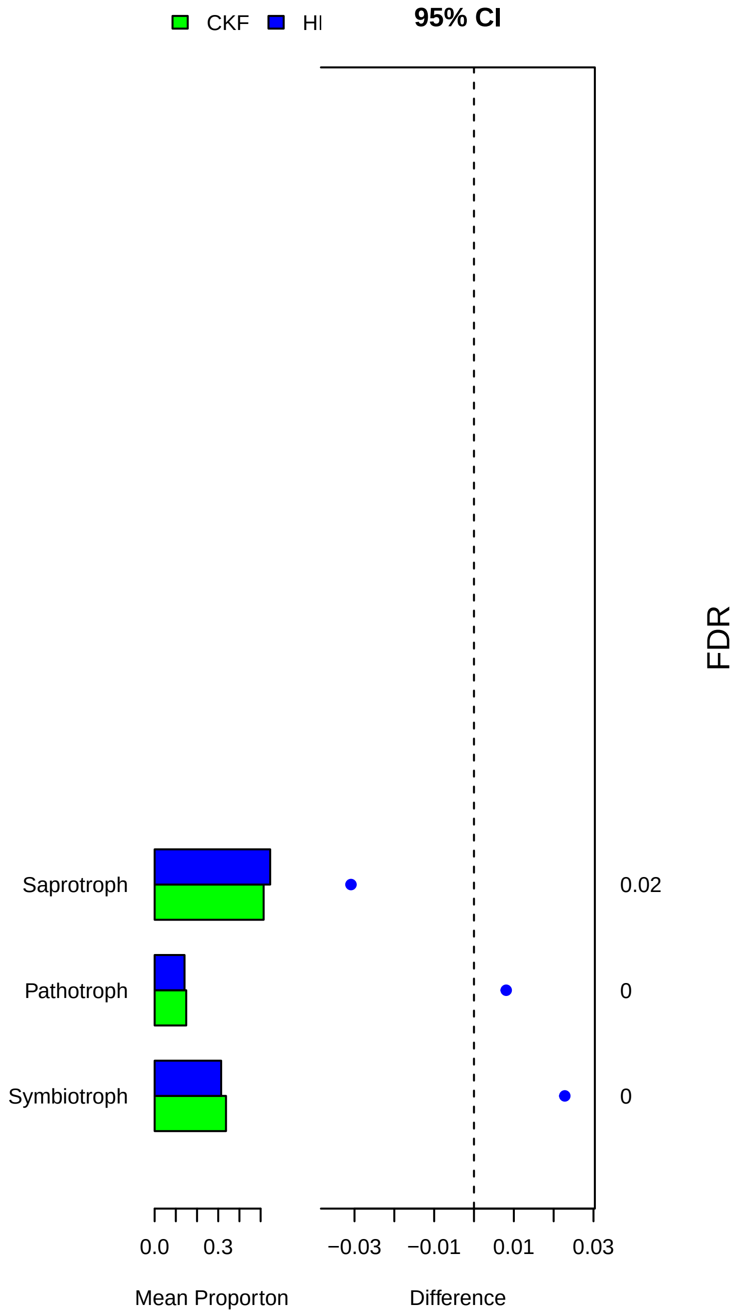
CKF
HI
95% CI
Saprotroph
Pathotroph
Symbiotroph
0.02
0
0
FDR
0.0
0.3
−0.03
−0.01
0.01
0.03
Mean Proporton
Difference
